# A Regression-Based Method for Estimating Risks and Relative Risks in Case-Base Studies

**DOI:** 10.1371/journal.pone.0083275

**Published:** 2013-12-12

**Authors:** Tina Tsz-Ting Chui, Wen-Chung Lee

**Affiliations:** 1 Institute of Epidemiology and Preventive Medicine, College of Public Health, National Taiwan University, Taipei, Taiwan; 2 Research Center for Genes, Environment and Human Health, College of Public Health, National Taiwan University, Taipei, Taiwan; University of Texas School of Public Health, United States of America

## Abstract

Both the absolute risk and the relative risk (RR) have a crucial role to play in epidemiology. RR is often approximated by odds ratio (OR) under the rare-disease assumption in conventional case-control study; however, such a study design does not provide an estimate for absolute risk. The case-base study is an alternative approach which readily produces RR estimation without resorting to the rare-disease assumption. However, previous researchers only considered one single dichotomous exposure and did not elaborate how absolute risks can be estimated in a case-base study. In this paper, the authors propose a logistic model for the case-base study. The model is flexible enough to admit multiple exposures in any measurement scale—binary, categorical or continuous. It can be easily fitted using common statistical packages. With one additional step of simple calculations of the model parameters, one readily obtains relative and absolute risk estimates as well as their confidence intervals. Monte-Carlo simulations show that the proposed method can produce unbiased estimates and adequate-coverage confidence intervals, for ORs, RRs and absolute risks. The case-base study with all its desirable properties and its methods of analysis fully developed in this paper may become a mainstay in epidemiology.

## Introduction

Both the absolute and the relative disease risks have a crucial role to play in epidemiology. The relative risk (RR) is the ratio of the disease risk for individuals at one specific exposure level to the disease risk for those at a reference level. Under the rare-disease assumption, RR is approximated by the odds ratio (OR), which in turn can be conveniently estimated in a case-control study. While an index such as RR or OR may be adequate for etiologic inferences, it is actually only part of a story. Once a factor has been demonstrated to be a risk factor for the disease, we will often be asked to predict the disease risk of an individual having a specific level of an exposure—the absolute risk. But unfortunately, the conventional case-control study does not provide an estimate for it.

Kupper et al [1] introduced a hybrid (part case-control, part cohort) design in a defined population (the ‘study base’)—the ‘case-base’ study later coined by Miettinen [2]. In contrast to the case-control study which samples the non-diseased subjects in the study base as the control group, the case-base study samples the entire study base with no regard to disease status. With such sampling scheme, the case-base study readily produces an RR estimate without resorting to the rare-disease assumption. Note that the case-base study should not be confused with the ‘case-cohort’ study introduced by Prentice [3]. The former, like the case-control study, is a *retrospective* design which ascertains the exposure statuses of subjects in a population retrospectively, while the latter is a *prospective* cohort study with all the time-to-event information available.

While the case-cohort study has been gaining popularity over the years [3]–[9], the case-base study remained little noticed since its introduction forty years ago. Miettinen [2] derived a variance formula for RR in a case-base study. Sato [10], [11] later proposed a more efficient estimator for RR, which is based on maximum likelihood estimation theory. However, these researchers only considered one dichotomous exposure and did not elaborate on how to estimate absolute risks in a case-base study. Without a general-purpose regression method for analyzing data, it is no wonder that most practicing epidemiologists would not consider the case-base design when planning a study.

In this paper, we develop a logistic model for the case-base study. The model is flexible enough to admit multiple exposures in any measurement scale—binary, categorical or continuous. It can be easily fitted using common statistical packages. With one additional step of simple calculations of the model parameters, one readily obtains relative and absolute risk estimates as well as their confidence intervals. We will use Monte-Carlo simulations to study the statistical properties of the proposed method.

## Methods

Let the exposure profile of a subject be denoted by a 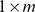 row vector 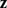. Each element of 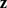 can be in either binary, categorical or continuous scale. Let 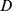 represents the disease status of a subject, with 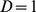 for diseased and 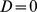 for non-diseased. We assume that the disease risk in the study population follows a logistic model:
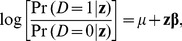
(1)


where 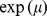 is the baseline disease odds (the disease odds for those with an exposure profile of 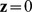 in the population) and 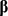 is a 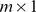 column vector of parameters of interest [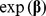 is a column vector of odds ratios].

In a case-base study, the ‘cases’ are randomly selected from all the incident diseased subjects in the population. Let 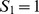 indicate that a diseased subject is recruited in the case sample, 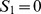, otherwise. Such a case sampling scheme implies that 
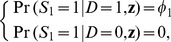
(2)


or more concisely, 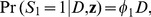
(3)


where 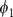 is a constant between 0 and 1. The ‘controls’ of a case-base study are randomly selected from all subjects in the population without regard to their disease status. Let 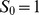 indicate that a subject is recruited in the control sample, 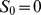, otherwise. Such a control sampling scheme implies that 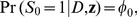
(4)


where 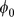 is a constant between 0 and 1. The two sampling schemes are independent to each other, that is,
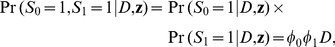
(5)


The event of 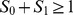 indicates that a subject is recruited in a case-base study through case sampling, control sampling or both. The recruitment probability of a subject with a disease status of 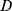 and an exposure profile of 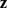 is
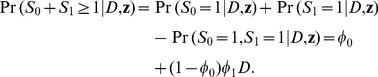
(6)


Let 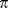 be the probability that a diseased subject in a case-base study is recruited in the control sample, that is, 
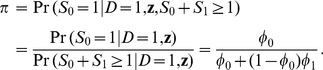
(7)



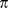
 is an important parameter to be used later.

From equations 1–7, we show below that the disease risk in a case-base sample also follows a logistic model as the one in the population (model 1), albeit with a different intercept:
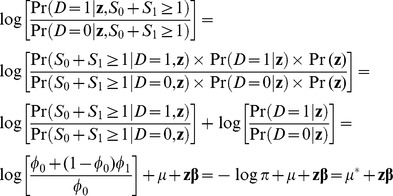
(8)


Suppose that there are a total of 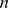 subjects recruited in a case-base study, who are indexed by 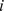 (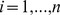). For the *i* ^th^ subject, the exposure profile, the disease status, and the control and the case sampling statuses are 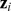, 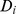, 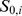, and 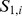, respectively. Given the exposure status of the subjects recruited in the case-base study, each of the subjects provides the information of disease and sampling statuses. The likelihood function is therefore
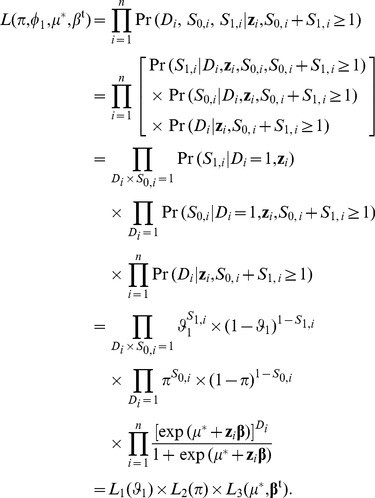
(9)


Because equation 9 is composed of three terms, the three sets of parameters (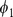 in 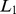, 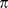 in 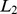, and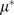 and 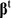 in 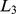) are mutually independent (the second derivatives of the log-likelihood with respect to parameters in different sets are zero).

Both 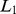 and 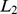 in equation 9 are binomial likelihoods. Therefore the maximum likelihood estimates of 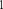 and 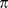, and their variances are: 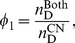
(10)

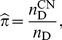
(11)

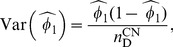
(12)


and 
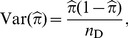
(13)


where 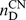 is the number of diseased subjects recruited in control sample, 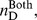 the number of diseased subjects recruited in both the case and the control sample, and 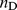, the total number of diseased subjects recruited in the case-base study.

The 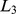 in equation 9 is a likelihood for a logistic regression model. To obtain the maximum likelihood estimates of 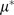
and 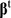, we can fit a logistic regression (model 8) to the case-base data. Note that the dependent variable of this logistic regression is the binary disease status with the diseased subjects coded as ‘1’ and the non-diseased subjects as ‘0’, regardless of their being recruited through case sampling, control sampling or both. Any statistical package that performs logistic regression analysis can obtain the estimates 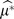
and 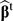, together with the variance-covariance matrix of (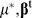). This variance-covariance matrix is denoted by 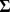, which is an 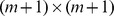 matrix.

The 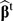 above readily provides the maximum likelihood estimates for the logarithms of ORs. As detailed below, the 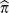 and 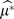 above are to be further combined to provide estimates for risks and RRs. First from model 8, an estimate for 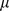 in model 1 is 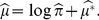
(14)


An estimate of the disease risk for subjects in the population with an exposure profile vector 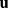 (a 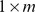 row vector ) is therefore
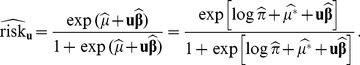
(15)


The variance of the estimate (in logit scale) is 
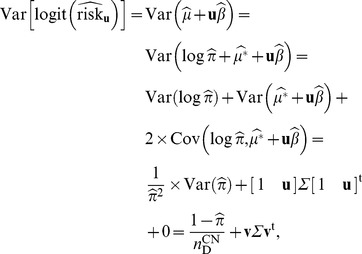
(16)


where 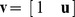 is a 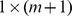 row vector. An estimate of the RR comparing those with an exposure profile vector 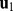 with those with 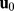 is 
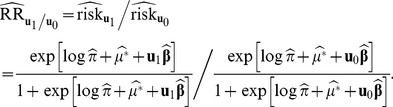
(17)


Using the delta method, the variance of the estimate (in log scale) is 
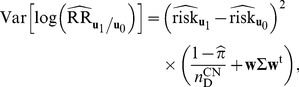
(18)


where 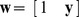 is a 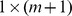 row vector with 
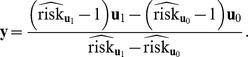


Exhibit S1 shows that Sato’s formulas [10], [11] of RR estimate and its variance in log scale are a special case of our formulas of equation 17 and 18 when there is only one single binary exposure.

Note that if 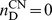 (no diseased subject is recruited in the control sample), 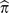 (in equation 11) is not estimable. Therefore, 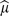 (in equation 14), 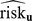 (in equation 15) and 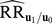
(in equation 17) are not estimable either. Under such setting, only the odds ratios, 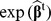, can be estimated in a case-base study. At the other extreme when 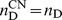 (all the diseased subjects are recruited in the control sample), we have 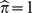 and 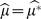, and therefore the case-base data can be analyzed as a cohort data. As for 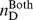 (number of diseased subject recruited in both the case and the control sample), if it is zero the 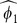 (in equation 10) is not estimable. This has no bearing whatsoever on the current context of estimating risks and relative risks however, since it is a nuisance parameter anyway.

We perform Monte-Carlo simulations to examine the statistical properties of the proposed method. We consider three scenarios for the exposure. In the first scenario, we assume a binary exposure (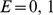). The exposure prevalence (for 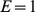) is set at 0.3. We assume that the OR comparing 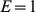 subjects with 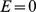 subjects is 2.5 (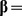
logOR = 0.9163). The disease prevalence in the study population is set at 0.1. Thus, the disease risk for 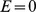 subjects (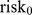) is 0.0727, the disease risk for 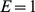 subjects (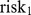) is 0.1638, and RR is 2.2543 (logRR = 0.8128).

In the second scenario, we assume an exposure with four levels (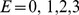). The exposure prevalence is set at 0.3 (for 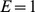), 0.1 (for 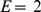), and 0.1 (for 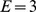), respectively. The OR comparing adjacent levels is set at 2.5 (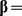
logOR = 0.9163). Again, we assume a disease prevalence of 0.1. Therefore, the four disease risks are 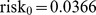, 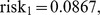 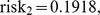 and 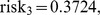 respectively, and the RRs are (with 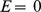 as the reference level) 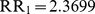 (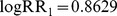), 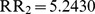 (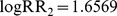), and 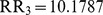 (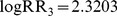), respectively.

In the third scenario, we assume two binary exposures (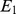 and 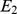). The exposure prevalence is set at 0.3 for 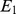, and 0.4 for 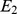. The OR comparing 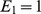 subjects with 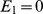 subjects is 2.5 (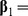
logOR_1_ = 0.9163), and the OR comparing 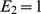 subjects with 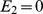 subjects is 3 (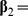
logOR_2_ = 1.0986). For simplicity, we assume that 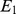 and 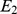 are independent of each other in the population and that there is no multiplicative interaction between 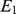 and 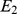 in causing the disease. The disease prevalence in the study population is set at 0.1. Thus, the four disease risks are 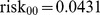 (for 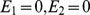), 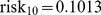 (for 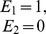), 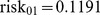 (for 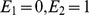), and 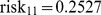 (for 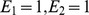), respectively. The RRs are (with 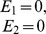 as the reference level) 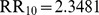 (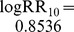),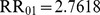 (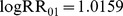), and 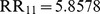 (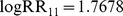), respectively.

The disease probabilities of subjects in the study population are assumed to follow the logistic model in model 1 with the parameter settings given in the preceding paragraphs. A case-base study is conducted in a study population of size 100000 with a case sampling probability (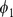) of 0.05 and a control sampling probability (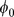) of 0.005. Under such sampling scheme, the case-base study is expected to recruit a total of 500 distinct diseased and 500 distinct non-diseased subjects. We use the proposed method to calculate the point estimates and 95 confidence intervals (CIs) for ORs, RRs and risks. For a comparison, Sato’s [10], [11] and Miettinen’s [2] methods are also performed.

The simulation was done for 10,000 times for each setting. The mean of the estimates for ORs (in log scale), RRs (in log scale) and risks (in logit scale) are calculated. The variance of an estimate is calculated as the sample variance of the estimates. We also calculate the coverage probability and the average length of the 95% CIs for the estimates.

## Results

Table 1 shows the simulation results for a binary exposure. For all methods, the RR estimates are approximately unbiased and the 95% CIs achieve adequate coverage probabilities. However, the variance and the length of 95% CIs for our method are much smaller than those for Miettinen’s methods. (Sato’s method for the case of one binary exposure is exactly the same as our method.) Only our method can produce estimates for OR and risks additionally. From Table 1, we see that these estimates are approximately unbiased and their 95% CIs achieve adequate coverage probabilities.

**Table 1:** Simulation results for a binary exposure.

	Methods			
	The present method	Sato	Miettinen	
Estimate [true value]				
logOR [0.9163]	0.9191	-	-	
logRR [0.8128]	0.8148	0.8149	0.8149	
logit(risk_0_) [–2.5465]	–2.5559	-	-	
logit(risk_1_) [–1.6303]	–1.6369	-	-	
Variance (×100)				
logOR	1.8297	-	-	
logRR	1.3984	1.3984	1.5017	
logit(risk_0_)	2.5622	-	-	
logit(risk_1_)	3.0710	-	-	
Coverage probability of 95% CI				
logOR	0.9521	-	-	
logRR	0.9518	0.9518	0.9518	
logit(risk_0_)	0.9512	-	-	
logit(risk_1_)	0.9497	-	-	
Average length of 95% CI				
logOR	0.5324	-	-	
logRR	0.4657	0.4657	0.4825	
logit(risk_0_)	0.6220	-	-	
logit(risk_1_)	0.6818	-	-	

Table 2 presents the simulation results for an exposure with four levels. It can be seen that our method can produce unbiased estimates and adequate-coverage 95% CIs for ORs, RRs, and risks. Sato’s and Miettinen’s methods can only produce estimates and 95% CIs for RRs. These two methods do not exploit the constancy in OR per unit change in the exposure variable. Therefore we see that though unbiased and with adequate coverage, they produce considerably larger variances and average length of 95% CIs as compared to our method. Exhibit S2 presents the simulation results for an exposure with four levels but without the constant OR assumption. We see that our method is still unbiased and with adequate coverage. The RR estimates are now the same as those using Sato’s method, though. Exhibit S3 shows that our method can produce unbiased estimates and adequate-coverage 95% CIs for ORs, RRs, and risks, when the exposure is in a continuous scale.

**Table 2:** Simulation results for an exposure with four levels.

	Methods		
	The present method	Sato	Miettinen
Estimate [true value]			
logOR comparing adjacent levels [0.9163]	0.9189	-	-
logRR_1_ [0.8629]	0.8655	0.8654	0.8654
logRR_2_ [1.6569]	1.6615	1.6648	1.6668
logRR_3_ [2.3203]	2.3253	2.3278	2.3297
logit(risk_0_) [–3.2708]	–3.2845	-	-
logit(risk_1_) [–2.3545]	–2.3656	-	-
logit(risk_2_) [–1.4383]	–1.4468	-	-
logit(risk_3_) [–0.5220]	–0.5279	-	-
Variance (×100)			
logOR comparing adjacent levels	0.4854	-	-
logRR_1_	0.4586	2.4588	2.5149
logRR_2_	1.5899	3.6685	4.0080
logRR_3_	2.6760	2.9777	3.4950
logit(risk_0_)	2.9127	-	-
logit(risk_1_)	2.3802	-	-
logit(risk_2_)	2.8184	-	-
logit(risk_3_)	4.2274	-	-
Coverage probability of 95% CI			
logOR comparing adjacent levels	0.9536	-	-
logRR_1_	0.9533	0.9563	0.9556
logRR_2_	0.9530	0.9487	0.9493
logRR_3_	0.9518	0.9526	0.9523
logit(risk_0_)	0.9518	-	-
logit(risk_1_)	0.9504	-	-
logit(risk_2_)	0.9505	-	-
logit(risk_3_)	0.9505	-	-
Average length of 95% CI			
logOR comparing adjacent levels	0.2731	-	-
logRR_1_	0.2657	0.6243	0.6319
logRR_2_	0.4952	0.7478	0.7814
logRR_3_	0.6437	0.6783	0.7330
logit(risk_0_)	0.6677	-	-
logit(risk_1_)	0.6011	-	-
logit(risk_2_)	0.6531	-	-
logit(risk_3_)	0.8007	-	-

Table 3 presents the simulation results for two binary exposures. Similarly, only our method can produce unbiased estimates and adequate-coverage 95% CIs for ORs, RRs, and risks. Sato’s and Miettinen’s methods can produce unbiased estimates and with adequate coverage 95% CIs for RRs only. These two methods do not exploit the assumption of no interaction between the two exposures. Therefore, we see that the variances and average length of 95% CIs for the two methods are much larger as compared to our method. Exhibit S4 presents the simulation results when there is an interaction effect between the two exposures. We see that our method can produce unbiased estimates and adequate-coverage 95% CIs for ORs, RRs, and risks, if an interaction term (cross-product term) is incorporated into the regression model. Exhibit S5 presents the simulation results for a confounder. We see that without adjusting for the confounder, one gets estimates that are biased and 95% CIs that are under-coverage. The problems can be easily fixed by performing a logistic regression analysis with both the study exposure and the confounder as its covariates.

**Table 3:** Simulation results for two binary exposures.

	Methods		
	The present method	Sato	Miettinen
Estimate [true value]			
logOR_1_ [0.9163]	0.9206	-	-
logOR_2_ [1.0986]	1.1017	-	-
logRR_10_ [0.8536]	0.8571	0.8580	0.8585
logRR_01_ [1.0159]	1.0184	1.0193	1.0197
logRR_11_ [1.7678]	1.7724	1.7741	1.7754
logit(risk_00_) [–3.0995]	–3.1087	-	-
logit(risk_10_) [–2.1832]	–2.1880	-	-
logit(risk_01_) [–2.0008]	–2.0070	-	-
logit(risk_11_) [–1.0846]	–1.0863	-	-
Variance (×100)			
logOR_1_	2.0187	-	-
logOR_2_	1.8573	-	-
logRR_10_	1.7228	3.2565	3.3754
logRR_01_	1.5893	2.4743	2.5707
logRR_11_	3.0231	3.0867	3.3906
logit(risk_00_)	3.1880	-	-
logit(risk_10_)	3.5971	-	-
logit(risk_01_)	3.0930	-	-
logit(risk_11_)	3.8039	-	-
Coverage probability of 95% CI			
logOR_1_	0.9490	-	-
logOR_2_	0.9503	-	-
logRR_10_	0.9492	0.9508	0.9509
logRR_01_	0.9508	0.9510	0.9486
logRR_11_	0.9484	0.9487	0.9532
logit(risk_00_)	0.9481	-	-
logit(risk_10_)	0.9470	-	-
logit(risk_01_)	0.9465	-	-
logit(risk_11_)	0.9487	-	-
Average length of 95% CI			
logOR_1_	0.5534	-	-
logOR_2_	0.5323	-	-
logRR_10_	0.5114	0.7034	0.7161
logRR_01_	0.4923	0.6149	0.6257
logRR_11_	0.6788	0.6862	0.7224
logit(risk_00_)	0.6875	-	-
logit(risk_10_)	0.7300	-	-
logit(risk_01_)	0.6767	-	-
logit(risk_11_)	0.7525	-	-

Exhibit S6 examines the situations when the disease prevalence is lower: 0.05 and 0.01, respectively. The conclusions about method comparisons remain the same, except that the precisions for RRs and risks are compromised across all methods.

## Discussion

Logistic regression is a standard technique for analyzing case-control data. It is also the method of choice for analyzing cohort data if time-to-event information is not available. However, the ORs that it estimates are approximating the RRs only under the rare-disease assumption. As such, there have been many methodologies/recommendations proposed to date regarding the estimation of RRs in cohort studies for common outcomes [12]–[17]. For example, Diaz-Quijano [17] described a novel regression-based method for estimating RRs in cohort studies. In his method, all the diseased subjects in the study are to be duplicated, and the duplicated subjects are to be re-labeled as the non-diseased. (For case-base studies, we can duplicate and re-label the diseased subjects recruited in the control sample.) Then, a logistic model is fitted to the expanded dataset, and the resulting regression coefficients are the estimates for logRRs. For case-base study, we found that such a data expansion approach produces an unbiased RR estimate for a binary exposure, but with a larger variance and a wider CI than our method; for a four-level exposure, the approach produces biased estimates and CIs with inadequate coverage (results not shown). For cohort study without time-to-event information, one can also apply our method to estimate ORs, RRs, and risks, except that the 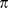 (equation 7) now is exactly one and is no longer a parameter to be estimated.

**Figure 1:**
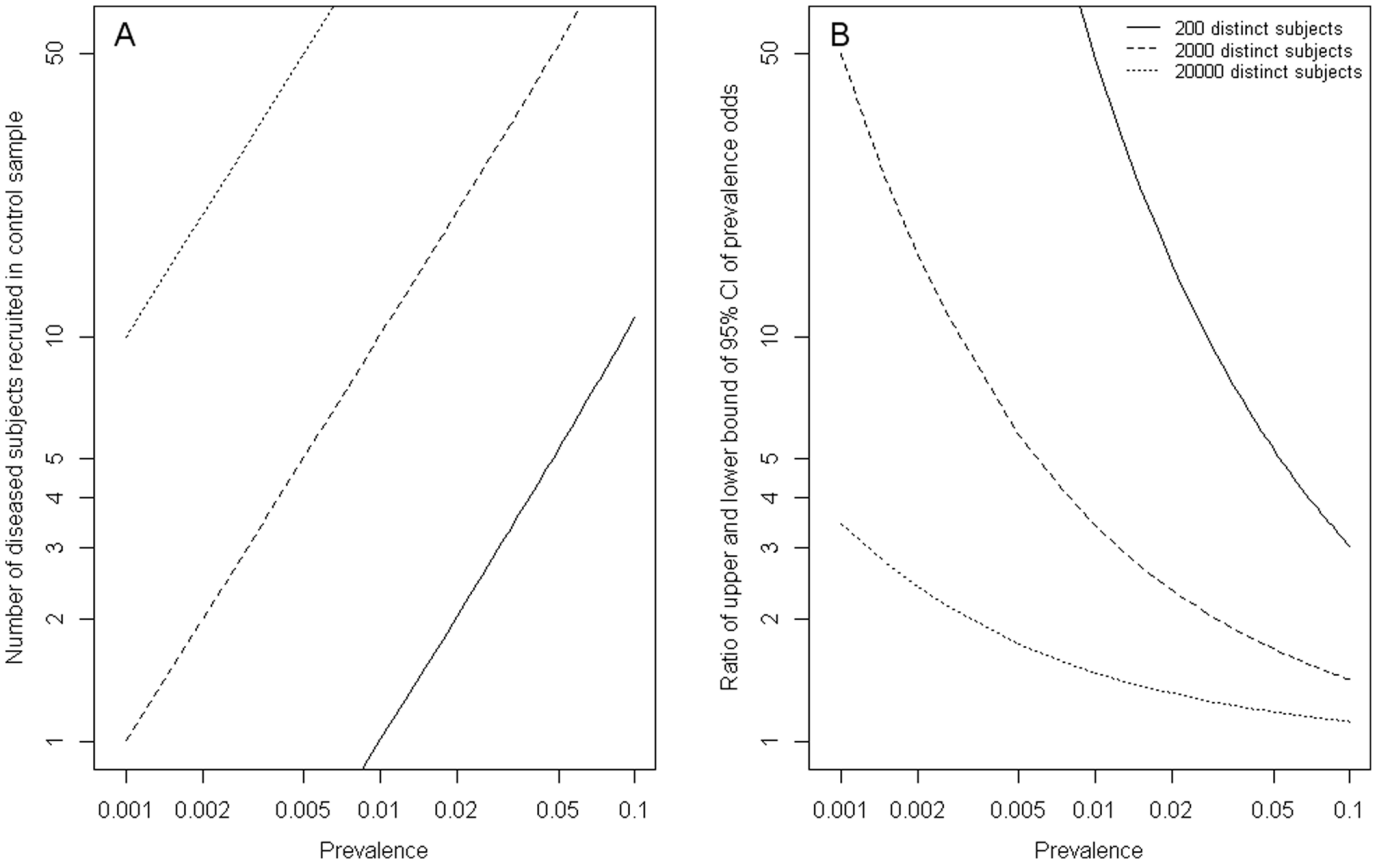
Number of diseased subjects recruited in control sample (A); Ratio of upper and lower bound of 95% confidence intervals of prevalence odds (B), in a case-base study of 200 distinct subjects (solid lines), 2000 distinct subjects (dashed lines) and 20000 distinct subjects (dotted lines).

In addition to the usual ORs, a case-base study also provides estimates for risks (equation 15) and RRs (equation 17). From equations 16 and 18, we see that the precision of the estimation is inversely proportional to 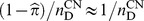, that is, the larger the 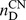 (number of diseased subjects recruited in control sample), the more precise the estimate of a risk or a RR. The value of 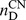 depends on the disease prevalence in the population and the sample size of the case-base study (Figure 1A). For a common disease (prevalence >0.05), a case-base study of 200 distinct subjects (with equal number of diseased and non-diseased subjects) is expected to have an 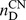 larger than 5, producing an estimate of disease odds with the upper 95% confidence bound being roughly 5 times its lower bound (Figure 1B). If the disease prevalence is lower (say, prevalence  = 0.005), one needs to increase the sample size of the case-base study (2000 subjects) to achieve comparable precision. If the registry system (for the diseased and the general population as well) in a population is readily available, the sample size then is no longer a limiting factor. In such setting, a case-base study can produce estimates for risks and RRs with reasonable precision, even if the disease is very rare (eg., 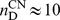 and 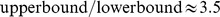 when sample size  = 20000 in a population with disease prevalence of 0.001).

In many respects, a case-base design is better than (or at least as good as) the commonly used case-control design. First, as just mentioned, a case-base study provides estimates not only for ORs but also for risks and RRs with reasonable accuracy (if 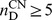). Second, the control sampling scheme of a case-base study is a simple random sampling of all subjects in the study population without regard to disease status. This means that a researcher can initiate the control recruitment process much earlier in a case-base design (at the outset of the study) than in a case-control design (at the end of the study). Third, although there could be some people sampled more than once in a case-base study, the sampling itself incurs minimal cost. The real cost constraint is usually the total number of *distinct* subjects that are actually recruited. And with the same total number of distinct subjects, a case-base study and a case-control study have exactly the same statistical efficiency, when it comes to estimating an OR. Finally, as shown in this study, the analysis of a case-base study is no more complicated than a case-control study—one needs only to fit a logistic regression model to the data and then do one extra step of simple calculations of the model parameters.

## Supporting Information

Exhibit S1 Comparison of Sato’s formulas and the formulas derived in this paper when there is only one single binary exposure.
(DOC)

Exhibit S2 Simulation results for an exposure with four levels but without the constant OR assumption.
(DOCX)

Exhibit S3 Simulation results when the exposure is in a continuous scale.
(DOCX)

Exhibit S4 Simulation results when there is an interaction effect between the two exposures.
(DOCX)

Exhibit S5 Simulation results for a confounder.
(DOCX)

Exhibit S6 Simulation results when the disease prevalence is lower.
(DOCX)
