# Supplementary material for: A Regression-Based Method for Estimating Risks and Relative Risks in Case-Base Studies
Source: PLoS One. 2013 Dec 12;8(12):e83275. doi: 10.1371/journal.pone.0083275 (PMC3861498; doi:10.1371/journal.pone.0083275)
Supplement: Exhibit S1 — Comparison of Sato’s formulas and the formulas derived in this paper when there is only one single binary exposure. (DOC) [file pone.0083275.s001.doc]

**Exhibit S1.** Comparison of Sato’s formulas and the formulas derived in this paper when there is only one single binary exposure.

Sato (10) used the following notations:

$(a_{0},b_{0})$ for the exposed ($a_{0}$) and the unexposed ($b_{0}$) diseased subjects recruited only in the case sample,

$(a_{1},b_{1})$ for the exposed ($a_{1}$) and the unexposed ($b_{1}$) diseased subjects recruited in both the case and control samples,

$(a_{2},b_{2})$ for the exposed ($a_{2}$) and the unexposed ($b_{2}$) diseased subjects recruited only in the control sample,

$(c,d)$ for the exposed ($c$) and the unexposed ($d$) non-diseased subjects recruited in the control sample,

$a_{+}=a_{0}+a_{1}+a_{2}$ for the total number of the exposed diseased subjects that are recruited,

$b_{+}=b_{0}+b_{1}+b_{2}$ for the total number of the unexposed diseased subjects that are recruited,

$e=a_{1}+a_{2}$ for the number of the exposed diseased subjects that are recruited in the control sample,

$f=b_{1}+b_{2}$ for the number of the unexposed diseased subjects that are recruited in the control sample,

$\tilde{e}=a_{+}\times(e+f)/(a_{+}+b_{+})$ for the expected number of the exposed diseased subjects that are recruited in the control sample,

$\tilde{f}=b_{+}\times(e+f)/(a_{+}+b_{+})$ for the expected number of the unexposed diseased subjects that are recruited in the control sample,

$\tilde{n}_{1}=\tilde{e}+c$ for the expected number of the exposed subjects that are recruited in the control sample,

$\tilde{n}_{0}=\tilde{f}+d$ for the expected number of the unexposed subjects that are recruited in the control sample,

and

$n=\tilde{n}_{1}+\tilde{n}_{0}$ for the total number of subjects in the control sample.

Based on Sato’s notations, we thus have ($E=1$ for exposed subjects; $E=0$ for unexposed subjects)

$a_{+}+b_{+}=n_{D}$,

from equation 7,

$(e+f)/(a_{+}+b_{+})=\widehat{\text{Pr}}\left(S_{0}=1|D=1,E,S_{0}+S_{1}\geq1\right)=\widehat{\pi}$,

$(a_{0}+b_{0})/\left[(a_{+}+b_{+})\times(e+f)\right]=(1-\widehat{\pi})/n_{\text{D}}^{\text{CN}}$,

from equation 8,

$$\begin{matrix} \left(\frac{e+f}{a_{+}+b_{+}}\right)\times\left(\frac{b_{+}}{d}\right)=\widehat{\pi}\times\widehat{\Pr}\left(D=1|E=0,S_{0}+S_{1}\geq1\right)/\widehat{\text{Pr}}\left(D=0|E=0,S_{0}+S_{1}\geq1\right) \\ \text{ } =\exp(\log\widehat{\pi}+\widehat{\mu^{*}}), \end{matrix}$$

and

$$\begin{matrix} \left(\frac{e+f}{a_{+}+b_{+}}\right)\times\left(\frac{a_{+}}{c}\right)=\widehat{\pi}\times\widehat{\Pr}\left(D=1|E=1,S_{0}+S_{1}\geq1\right)/\widehat{\text{Pr}}\left(D=0|E=1,S_{0}+S_{1}\geq1\right) \\ \text{ } \text{ }=\exp(\log\widehat{\pi}+\widehat{\mu^{*}}+\widehat{\beta}). \end{matrix}$$

Sato’s formula of relative risk estimation is

$$\begin{matrix} \widehat{\text{RR}}_{E=1/E=0}=\left(\frac{a_{+}}{\tilde{n}_{1}}\right)\times\left(\frac{b_{+}}{\tilde{n}_{0}}\right)^{-1} \\ =\left[\frac{\left(\frac{e+f}{a_{+}+b_{+}}\right)\times\frac{a_{+}}{c}}{\left(\frac{e+f}{a_{+}+b_{+}}\right)\times\frac{a_{+}}{c}+1}\right]\times\left[\frac{\left(\frac{e+f}{a_{+}+b_{+}}\right)\times\frac{b_{+}}{d}}{\left(\frac{e+f}{a_{+}+b_{+}}\right)\times\frac{b_{+}}{d}+1}\right]^{-1} \\ =\frac{\exp(\log\widehat{\pi}+\widehat{\mu^{*}}+\widehat{\beta})}{1+\exp(\log\widehat{\pi}+\widehat{\mu^{*}}+\widehat{\beta})}/\frac{\exp(\log\widehat{\pi}+\widehat{\mu^{*}})}{1+\exp(\log\widehat{\pi}+\widehat{\mu^{*}})}, \end{matrix}$$

which is the equation 17 of our formula.

Sato’s formula of the variance of the log relative risk is

$$\begin{matrix} \text{Var}\left[\log\left(\widehat{\text{RR}}_{E=1/E=0}\right)\right] \\ \text{ }=\frac{1}{a_{+}}+\frac{1}{b_{+}}+\left(1-2\times\frac{e+f}{a_{+}+b_{+}}\right)\times\left(\frac{1}{\tilde{n}_{1}}+\frac{1}{\tilde{n}_{0}}\right)-\frac{n^{2}a_{+}b_{+}(a_{0}+b_{0})(e+f)}{(a_{+}+b_{+})^{3}\tilde{n}_{1}^{2}\tilde{n}_{0}^{2}} \end{matrix}$$

$$=\left(\frac{\tilde{e}}{\tilde{n}_{1}}-\frac{\tilde{f}}{\tilde{n}_{0}}\right)^{2}\times\left[\frac{a_{0}+b_{0}}{(a_{+}+b_{+})(e+f)}+\frac{1}{b_{+}}+\frac{1}{d}\right]+\left(\frac{\tilde{e}}{\tilde{n}_{1}}-1\right)^{2}\times\left[\left(\frac{1}{a_{+}}+\frac{1}{c}\right)+\left(\frac{1}{b_{+}}+\frac{1}{d}\right)\right]$$

$$-2\times\left(\frac{\tilde{e}}{\tilde{n}_{1}}-1\right)\times\left(\frac{\tilde{e}}{\tilde{n}_{1}}-\frac{\tilde{f}}{\tilde{n}_{0}}\right)\times\left(\frac{1}{b_{+}}+\frac{1}{d}\right)$$

$$\begin{matrix} =\left(\widehat{\text{risk}}_{E=1}-\widehat{\text{risk}}_{E=0}\right)^{2}\times\left[\frac{(1-\widehat{\pi})}{n_{\text{D}}^{\text{CN}}}+\text{Var}\left(\widehat{\mu^{*}}\right)\right]+\left(\widehat{\text{risk}}_{E=1}-1\right)^{2}\times\text{Var(}\widehat{\beta}) \\ +2\times\left(\widehat{\text{risk}}_{E=1}-1\right)\times\left(\widehat{\text{risk}}_{E=1}-\widehat{\text{risk}}_{E=0}\right)\times\text{Cov}\left(\widehat{\mu^{*}},\widehat{\beta}\right) \end{matrix}$$

$$=\left(\widehat{\text{risk}}_{E=1}-\widehat{\text{risk}}_{E=0}\right)^{2}\times\left[\frac{(1-\widehat{\pi})}{n_{\text{D}}^{\text{CN}}}+\text{Var}\left(\widehat{\mu^{*}}\right)+y^{2}\times\text{Var(}\widehat{\beta})+2y\times\text{Cov}\left(\widehat{\mu^{*}},\widehat{\beta}\right)\right],$$

which is the equation 18 of our formula, where the risks for the exposed and the unexposed are, respectively,

$$\widehat{\text{risk}}_{E=1}=\frac{\exp(\log\widehat{\pi}+\widehat{\mu^{*}}+\widehat{\beta})}{1+\exp(\log\widehat{\pi}+\widehat{\mu^{*}}+\widehat{\beta})}=\frac{a_{+}\times\left(\frac{e+f}{a_{+}+b_{+}}\right)}{a_{+}\times\left(\frac{e+f}{a_{+}+b_{+}}\right)+c}=\frac{\tilde{e}}{\tilde{n}_{1}},$$

and

$$\widehat{\text{risk}}_{E=0}=\frac{\exp(\log\widehat{\pi}+\widehat{\mu^{*}})}{1+\exp(\log\widehat{\pi}+\widehat{\mu^{*}})}=\frac{b_{+}\times\left(\frac{e+f}{a_{+}+b_{+}}\right)}{b_{+}\times\left(\frac{e+f}{a_{+}+b_{+}}\right)+d}=\frac{\tilde{f}}{\tilde{n}_{0}},$$

and the variances of and the covariance between $\widehat{\mu^{*}}$ and $\widehat{\beta}$ can be shown to be

$\text{Var}\left(\widehat{\mu^{*}}\right)=\frac{1}{b_{+}}+\frac{1}{d}$,

$\text{Var(}\widehat{\beta})=\left(\frac{1}{a_{+}}+\frac{1}{c}\right)+\left(\frac{1}{b_{+}}+\frac{1}{d}\right)$,

and

$$\text{Cov}\left(\widehat{\mu^{*}},\widehat{\beta}\right)=-\left(\frac{1}{b_{+}}+\frac{1}{d}\right),$$

respectively.
