# Supplementary material for: A Regression-Based Method for Estimating Risks and Relative Risks in Case-Base Studies
Source: PLoS One. 2013 Dec 12;8(12):e83275. doi: 10.1371/journal.pone.0083275 (PMC3861498; doi:10.1371/journal.pone.0083275)
Supplement: Exhibit S2 — Simulation results for an exposure with four levels but without the constant OR assumption. (DOCX) [file pone.0083275.s002.docx]

**Exhibit S2.** Simulation results for an exposure with four levels but without the constant OR assumption.

Here, we examine the situations when the odds ratio (OR) comparing adjacent levels is not equal. Other settings are the same as in the text. A total of 10000 simulations are done for each scenario. The results are shown below:

| An exposure with four levels where the OR comparing adjacent levels is not equal | Methods | | | |
| --- | --- | --- | --- | --- |
|  | The present method | | Sato | Miettinen |
| Estimate [true value] |  | |  |  |
| logOR_1_  [0.9163] | 0.9187 | | - | - |
| logOR_2_  [1.0986] | 1.1076 | | - | - |
| logOR_3_  [1.7048] | 1.7154 | | - | - |
| logRR_1_ [0.8408] | 0.8429 | | 0.8429 | 0.8429 |
| logRR_2_ [0.9991] | 1.0054 | | 1.0054 | 1.0066 |
| logRR_3_ [1.4934] | 1.5000 | | 1.5000 | 1.5019 |
| logit(risk_0_) [-2.8972] | -2.9113 | | - | - |
| logit(risk_1_) [-1.9809] | -1.9926 | | - | - |
| logit(risk_2_) [-1.7985] | -1.8037 | | - | - |
| logit(risk_3_) [-1.1924] | -1.1959 | | - | - |
| Variance ($\times100$) |  | |  |  |
| logOR_1_ | 2.3837 | | - | - |
| logOR_2_ | 4.8806 | | - | - |
| logOR_3_ | 4.6053 | | - | - |
| logRR_1_ | 1.9921 | | 1.9921 | 2.0796 |
| logRR_2_ | 3.7743 | | 3.7744 | 4.0933 |
| logRR_3_ | 3.0813 | | 3.0814 | 3.4800 |
| logit(risk_0_) | 3.0629 | | - | - |
| logit(risk_1_) | 3.2021 | | - | - |
| logit(risk_2_) | 5.6525 | | - | - |
| logit(risk_3_) | 5.5096 | | - | - |
| Coverage probability of 95% CI | |  | |  |
| logOR_1_ | 0.9495 | | - | - |
| logOR_2_ | 0.9520 | | - | - |
| logOR_3_ | 0.9521 | | - | - |
| logRR_1_ | 0.9489 | | 0.9489 | 0.9482 |
| logRR_2_ | 0.9525 | | 0.9525 | 0.9491 |
| logRR_3_ | 0.9524 | | 0.9524 | 0.9552 |
| logit(risk_0_) | 0.9467 | | - | - |
| logit(risk_1_) | 0.9511 | | - | - |
| logit(risk_2_) | 0.9483 | | - | - |
| logit(risk_3_) | 0.9478 | | - | - |
| Average length of 95% CI | |  | |  |
| logOR_1_ | 0.6066 | | - | - |
| logOR_2_ | 0.8600 | | - | - |
| logOR_3_ | 0.8394 | | - | - |
| logRR_1_ | 0.5543 | | 0.5543 | 0.5659 |
| logRR_2_ | 0.7567 | | 0.7567 | 0.7842 |
| logRR_3_ | 0.6891 | | 0.6891 | 0.7323 |
| logit(risk_0_) | 0.6771 | | - | - |
| logit(risk_1_) | 0.6929 | | - | - |
| logit(risk_2_) | 0.9232 | | - | - |
| logit(risk_3_) | 0.9041 | | - | - |
