# Supplementary material for: A Regression-Based Method for Estimating Risks and Relative Risks in Case-Base Studies
Source: PLoS One. 2013 Dec 12;8(12):e83275. doi: 10.1371/journal.pone.0083275 (PMC3861498; doi:10.1371/journal.pone.0083275)
Supplement: Exhibit S3 — Simulation results when the exposure is in a continuous scale. (DOCX) [file pone.0083275.s003.docx]

**Exhibit S3.** Simulation results when the exposure is in a continuous scale.

Here, we examine the case of a continuous exposure. We assume that the exposure (*E*) is a continuous variable uniformly distributed between 0 and 1. Also, we assume the disease risk in the study population follows a logistic model:

$$\text{logit}\left(\text{risk}_{E=e}\right)=-2.6883+0.9163\times e.$$

Other settings are the same as in the text. The estimates of OR, RR and logit(risk) of $E=0.25,$0.50, 0.75 and 1.00 with a reference level $E=0.00$ are calculated. A total of 10000 simulations are performed for each scenario. The results are shown below:

| Continuous exposure |  | The present method | | | |
| --- | --- | --- | --- | --- | --- |
|  | True value | Estimate | Variance ($\times100$) | Coverage probability of 95% CI | Average length of 95% CI |
| logOR_0.25_ | 0.2291 | 0.2302 | 0.3151 | 0.9509 | 0.2204 |
| logOR_0.50_ | 0.4582 | 0.4605 | 1.2605 | 0.9509 | 0.4408 |
| logOR_0.75_ | 0.6872 | 0.6907 | 2.8360 | 0.9509 | 0.6612 |
| logOR_1.00_ | 0.9163 | 0.9209 | 5.0418 | 0.9509 | 0.8816 |
| logRR_0.25_ | 0.2128 | 0.2142 | 0.2862 | 0.9504 | 0.2101 |
| logRR_0.50_ | 0.4218 | 0.4244 | 1.1099 | 0.9505 | 0.4139 |
| logRR_0.75_ | 0.6262 | 0.6296 | 2.4017 | 0.9508 | 0.6089 |
| logRR_1.00_ | 0.8251 | 0.8289 | 4.0664 | 0.9510 | 0.7925 |
| logit(risk_0.00_) | -2.6883 | -2.6991 | 3.6379 | 0.9541 | 0.7522 |
| logit(risk_0.25_) | -2.4593 | -2.4688 | 2.6483 | 0.9547 | 0.6397 |
| logit(risk_0.50_) | -2.2302 | -2.2386 | 2.2890 | 0.9510 | 0.5914 |
| logit(risk_0.75_) | -2.0011 | -2.0084 | 2.5599 | 0.9521 | 0.6228 |
| logit(risk_1.00_) | -1.7720 | -1.7781 | 3.4610 | 0.9511 | 0.7234 |
