# Supplementary material for: A Regression-Based Method for Estimating Risks and Relative Risks in Case-Base Studies
Source: PLoS One. 2013 Dec 12;8(12):e83275. doi: 10.1371/journal.pone.0083275 (PMC3861498; doi:10.1371/journal.pone.0083275)
Supplement: Exhibit S4 — Simulation results when there is an interaction effect between the two exposures. (DOCX) [file pone.0083275.s004.docx]

**Exhibit S4.**  Simulation results when there is an interaction effect between the two exposures.

Here, we examine the situations when there is an interaction effect between the two exposures. Other settings are the same as in the text. A total of 10000 simulations are done for each scenario. We compare the analyses with and without the interaction. The results are shown below:

| Two binary exposures with interaction |  | Methods | |
| --- | --- | --- | --- |
|  | True value | With interaction term | Without interaction term |
| Estimate |  |  |  |
| logOR_1_ | 0.9163 | 0.9201 | 1.1192 |
| logOR_2_ | 1.0986 | 1.1008 | 1.2531 |
| logOR_3_ | 2.4204 | 2.4335 | 2.3723 |
| logRR_10_ | 0.8600 | 0.8630 | 1.0478 |
| logRR_01_ | 1.0243 | 1.0260 | 1.1675 |
| logRR_11_ | 2.0872 | 2.0944 | 2.0742 |
| logit(risk_00_) | -3.2159 | -3.2256 | -3.3075 |
| logit(risk_10_) | -2.2997 | -2.3055 | -2.1883 |
| logit(risk_01_) | -2.1173 | -2.1249 | -2.0544 |
| logit(risk_11_) | -0.7956 | -0.7922 | -0.9352 |
| Variance ($\times100$) |  |  |  |
| logOR_1_ |  | 3.9386 | 1.9892 |
| logOR_2_ |  | 3.1086 | 1.9782 |
| logOR_3_ |  | 4.7305 | 4.2424 |
| logRR_10_ |  | 3.4208 | 1.7223 |
| logRR_01_ |  | 2.7020 | 1.7276 |
| logRR_11_ |  | 3.1355 | 3.0870 |
| logit(risk_00_) |  | 3.5761 | 3.3076 |
| logit(risk_10_) |  | 4.1935 | 3.5413 |
| logit(risk_01_) |  | 3.2618 | 3.0315 |
| logit(risk_11_) |  | 4.8589 | 3.5401 |
| Coverage probability of 95% CI |  | |  |
| logOR_1_ |  | 0.9540 | 0.7161 |
| logOR_2_ |  | 0.9486 | 0.7992 |
| logOR_3_ |  | 0.9499 | 0.9422 |
| logRR_10_ |  | 0.9539 | 0.7161 |
| logRR_01_ |  | 0.9485 | 0.8025 |
| logRR_11_ |  | 0.9478 | 0.9491 |
| logit(risk_00_) |  | 0.9504 | 0.9255 |
| logit(risk_10_) |  | 0.9527 | 0.9013 |
| logit(risk_01_) |  | 0.9512 | 0.9306 |
| logit(risk_11_) |  | 0.9529 | 0.9010 |
| Average length of 95% CI |  | |  |
| logOR_1_ |  | 0.7839 | 0.5608 |
| logOR_2_ |  | 0.6851 | 0.5448 |
| logOR_3_ |  | 0.8411 | 0.8086 |
| logRR_10_ |  | 0.7297 | 0.5208 |
| logRR_01_ |  | 0.6381 | 0.5080 |
| logRR_11_ |  | 0.6836 | 0.6846 |
| logit(risk_00_) |  | 0.7318 | 0.7000 |
| logit(risk_10_) |  | 0.8044 | 0.7370 |
| logit(risk_01_) |  | 0.7084 | 0.6822 |
| logit(risk_11_) |  | 0.8603 | 0.7493 |
