# Supplementary material for: A Regression-Based Method for Estimating Risks and Relative Risks in Case-Base Studies
Source: PLoS One. 2013 Dec 12;8(12):e83275. doi: 10.1371/journal.pone.0083275 (PMC3861498; doi:10.1371/journal.pone.0083275)
Supplement: Exhibit S5 — Simulation results for a confounder. (DOCX) [file pone.0083275.s005.docx]

**Exhibit S5.** Simulation results for a confounder.

Here, we use regression analysis to adjust for confounding factors. We assume a binary exposure (*E* = 0 or 1) with exposure prevalence set at 0.3. In addition, we assume a binary confounder (*C* = 0 or 1) with the prevalence of *C* = 1 set at 0.6. The *E* and the *C* are assumed to be correlated in the study population with an odds ratio of 1.5. We assume the disease risk in the study population follows a logistic model:

$$\text{logit}\left(\text{risk}_{E=e,C=c}\right)=-2.9480+0.9163\times e+0.6\times c.$$

Other settings are the same as in the text. We compare the analyses with and without the adjustment of confounding. A total of 10000 simulations are done for each scenario. The results are shown below:

| Binary exposure with confounding |  | Methods | |
| --- | --- | --- | --- |
|  | True value | With adjustment | Without adjustment |
| Estimate |  |  |  |
| logOR | 0.9163 | 0.9185 | 0.9640 |
| logRR |  |  |  |
| *C*=0 | 0.8442 | 0.8459 | 0.8544 |
| *C*=1 | 0.7933 | 0.7948 | 0.8544 |
| logit(risk_0_) |  |  |  |
| *C*=0 | -2.9480 | -2.9869 | -2.5786 |
| *C*=1 | -2.3480 | -2.3616 | -2.5786 |
| logit(risk_1_) |  |  |  |
| *C*=0 | -2.0317 | -2.0684 | -1.6146 |
| *C*=1 | -1.4317 | -1.4431 | -1.6146 |
| Variance ($\times100$) |  |  |  |
| logOR |  | 1.8697 | 1.8139 |
| logRR |  |  |  |
| *C*=0 |  | 1.5677 | 1.3786 |
| *C*=1 |  | 1.3675 | 1.3786 |
| logit(risk_0_) |  |  |  |
| *C*=0 |  | 9.5597 | 2.5516 |
| *C*=1 |  | 3.4820 | 2.5516 |
| logit(risk_1_) |  |  |  |
| *C*=0 |  | 10.2337 | 3.1347 |
| *C*=1 |  | 4.0426 | 3.1347 |
| Coverage probability of 95% CI |  | |  |
| logOR |  | 0.9520 | 0.9408 |
| logRR |  |  |  |
| *C*=0 |  | 0.9514 | 0.9533 |
| *C*=1 |  | 0.9527 | 0.9263 |
| logit(risk_0_) |  |  |  |
| *C*=0 |  | 0.9525 | 0.3593 |
| *C*=1 |  | 0.9550 | 0.7171 |
| logit(risk_1_) |  |  |  |
| *C*=0 |  | 0.9522 | 0.3299 |
| *C*=1 |  | 0.9514 | 0.8330 |
| Average length of 95% CI |  | |  |
| logOR |  | 0.5391 | 0.5330 |
| logRR |  |  |  |
| *C*=0 |  | 0.4942 | 0.4659 |
| *C*=1 |  | 0.4628 | 0.4659 |
| logit(risk_0_) |  |  |  |
| *C*=0 |  | 1.1671 | 0.6236 |
| *C*=1 |  | 0.7316 | 0.6236 |
| logit(risk_1_) |  |  |  |
| *C*=0 |  | 1.2086 | 0.6821 |
| *C*=1 |  | 0.7765 | 0.6821 |
