# Supplementary material for: A Regression-Based Method for Estimating Risks and Relative Risks in Case-Base Studies
Source: PLoS One. 2013 Dec 12;8(12):e83275. doi: 10.1371/journal.pone.0083275 (PMC3861498; doi:10.1371/journal.pone.0083275)
Supplement: Exhibit S6 — Simulation results when the disease prevalence is lower. (DOCX) [file pone.0083275.s006.docx]

**Exhibit S6.**  Simulation results when the disease prevalence is lower.

In text, the disease prevalence is set at 0.1. Here, we examine the situations when the disease prevalence is lower: 0.05 and 0.01, respectively. The case sampling probability ($\varphi_{1}$) varies with the disease prevalence to recruit an expected number of 500 distinct diseased subjects. All other parameters are the same as in the text. The simulation results are shown below:

| A binary exposure with the disease prevalence=0.05 | Methods | | | | | | | |  |
| --- | --- | --- | --- | --- | --- | --- | --- | --- | --- |
|  | The present method | | Sato | | | Miettinen | |  |  |
| Estimate [true value] |  | | |  | | |  | |  |
| logOR [0.9163] | 0.9193 | | | - | | | - | |  |
| logRR [0.8645] | 0.8671 | | | 0.8671 | | | 0.8668 | |  |
| logit(risk_0_) [-3.3048] | -3.3245 | | | - | | | - | |  |
| logit(risk_1_) [-2.3885] | -2.4052 | | | - | | | - | |  |
| Variance ($\times100$) |  | | |  | | |  | |  |
| logOR | 1.8330 | | | - | | | - | |  |
| logRR | 1.6098 | | | 1.6105 | | | 1.6769 | |  |
| logit(risk_0_) | 4.6515 | | | - | | | - | |  |
| logit(risk_1_) | 5.0986 | | | - | | | - | |  |
| Coverage probability of 95% CI | |  | | |  | | | | |
| logOR | 0.9471 | | | - | | | - | |  |
| logRR | 0.9473 | | | 0.9472 | | | 0.9477 | |  |
| logit(risk_0_) | 0.9559 | | | - | | | - | |  |
| logit(risk_1_) | 0.9573 | | | - | | | - | |  |
| Average length of 95% CI | |  | | |  | | | | |
| logOR | 0.5255 | | | - | | | - | |  |
| logRR | 0.4926 | | | 0.4926 | | | 0.5010 | |  |
| logit(risk_0_) | 0.8423 | | | - | | | - | |  |
| logit(risk_1_) | 0.8808 | | | - | | | - | |  |

| A binary exposure with the disease prevalence=0.01 | Methods | | | | | | | |  |
| --- | --- | --- | --- | --- | --- | --- | --- | --- | --- |
|  | The present method | | Sato | | | Miettinen | |  |  |
| Estimate [true value] |  | | |  | | |  | |  |
| logOR [0.9163] | 0.9193 | | | - | | | - | |  |
| logRR [0.9059] | 0.9089 | | | 0.9089 | | | 0.9090 | |  |
| logit(risk_0_) [-4.9644] | -5.0656 | | | - | | | - | |  |
| logit(risk_1_) [-4.0482] | -4.1462 | | | - | | | - | |  |
| Variance ($\times100$) |  | | |  | | |  | |  |
| logOR | 1.7578 | | | - | | | - | |  |
| logRR | 1.7190 | | | 1.7271 | | | 1.7282 | |  |
| logit(risk_0_) | 2.6228 | | | - | | | - | |  |
| logit(risk_1_) | 2.6388 | | | - | | | - | |  |
| Coverage probability of 95% CI | |  | | |  | | | | |
| logOR | 0.9512 | | | - | | | - | |  |
| logRR | 0.9521 | | | 0.9523 | | | 0.9523 | |  |
| logit(risk_0_) | 0.9672 | | | - | | | - | |  |
| logit(risk_1_) | 0.9657 | | | - | | | - | |  |
| Average length of 95% CI | |  | | |  | | | | |
| logOR | 0.5216 | | | - | | | - | |  |
| logRR | 0.5150 | | | 0.5151 | | | 0.5168 | |  |
| logit(risk_0_) | 1.9295 | | | - | | | - | |  |
| logit(risk_1_) | 1.9451 | | | - | | | - | |  |

*The estimations of logRR, logit(risk_0_) and logit(risk_1_) of our method are based on 9929 simulations. All the other parameters are based on 10000 simulations.

| An exposure with four levels with the disease prevalence=0.05 | Methods | | | |
| --- | --- | --- | --- | --- |
|  | The present method | | Sato | Miettinen |
| Estimate [true value] |  | |  |  |
| logOR comparing adjacent levels [0.9163] | 0.9190 | | - | - |
| logRR_1_ [0.8921] | 0.8948 | | 0.8929 | 0.8929 |
| logRR_2_ [1.7505] | 1.7554 | | 1.7567 | 1.7579 |
| logRR_3_ [2.5350] | 2.5412 | | 2.5449 | 2.5466 |
| logit(risk_0_) [-4.0999] | -4.1222 | | - | - |
| logit(risk_1_) [-3.1836] | -3.2032 | | - | - |
| logit(risk_2_) [-2.2673] | -2.2842 | | - | - |
| logit(risk_3_) [-1.3510] | -1.3652 | | - | - |
| Variance ($\times100$) |  | |  |  |
| logOR comparing adjacent levels | 0.4619 | | - | - |
| logRR_1_ | 0.4531 | | 2.8903 | 2.9251 |
| logRR_2_ | 1.6991 | | 4.2806 | 4.4562 |
| logRR_3_ | 3.2895 | | 3.6941 | 3.9789 |
| logit(risk_0_) | 5.1987 | | - | - |
| logit(risk_1_) | 4.5637 | | - | - |
| logit(risk_2_) | 4.8526 | | - | - |
| logit(risk_3_) | 6.0653 | | - | - |
| Coverage probability of 95% CI | |  | |  |
| logOR comparing adjacent levels | 0.9500 | | - | - |
| logRR_1_ | 0.9517 | | 0.9534 | 0.9532 |
| logRR_2_ | 0.9517 | | 0.9498 | 0.9497 |
| logRR_3_ | 0.9508 | | 0.9485 | 0.9510 |
| logit(risk_0_) | 0.9520 | | - | - |
| logit(risk_1_) | 0.9514 | | - | - |
| logit(risk_2_) | 0.9519 | | - | - |
| logit(risk_3_) | 0.9504 | | - | - |
| Average length of 95% CI | |  | |  |
| logOR comparing adjacent levels | 0.2641 | | - | - |
| logRR_1_ | 0.2616 | | 0.6714 | 0.6748 |
| logRR_2_ | 0.5066 | | 0.8084 | 0.8246 |
| logRR_3_ | 0.7050 | | 0.7426 | 0.7704 |
| logit(risk_0_) | 0.8840 | | - | - |
| logit(risk_1_) | 0.8284 | | - | - |
| logit(risk_2_) | 0.8555 | | - | - |
| logit(risk_3_) | 0.9579 | | - | - |

| An exposure with four levels with the disease prevalence=0.01 | Methods | | | |
| --- | --- | --- | --- | --- |
|  | The present method | | Sato | Miettinen |
| Estimate [true value] |  | |  |  |
| logOR comparing adjacent levels [0.9163] | 0.9197 | | - | - |
| logRR_1_ [0.9118] | 0.9152 | | 0.9155 | 0.9156 |
| logRR_2_ [1.8171] | 1.8238 | | 1.8276 | 1.8276 |
| logRR_3_ [2.7063] | 2.7158 | | 2.7201 | 2.7204 |
| logit(risk_0_) [-5.8138] | -5.9239 | | - | - |
| logit(risk_1_) [-4.8975] | -5.0042 | | - | - |
| logit(risk_2_) [-3.9813] | -4.0845 | | - | - |
| logit(risk_3_) [-3.0650] | -3.1648 | | - | - |
| Variance ($\times100$) |  | |  |  |
| logOR comparing adjacent levels | 0.4352 | | - | - |
| logRR_1_ | 0.4343 | | 3.2702 | 3.2727 |
| logRR_2_ | 1.7162 | | 4.8158 | 4.8357 |
| logRR_3_ | 3.7401 | | 4.1913 | 4.2769 |
| logit(risk_0_) | 26.5565 | | - | - |
| logit(risk_1_) | 25.9227 | | - | - |
| logit(risk_2_) | 26.1600 | | - | - |
| logit(risk_3_) | 27.2684 | | - | - |
| Coverage probability of 95% CI | |  | |  |
| logOR comparing adjacent levels | 0.9509 | | - | - |
| logRR_1_ | 0.9499 | | 0.9515 | 0.9518 |
| logRR_2_ | 0.9501 | | 0.9536 | 0.9522 |
| logRR_3_ | 0.9488 | | 0.9491 | 0.9467 |
| logit(risk_0_) | 0.9661 | | - | - |
| logit(risk_1_) | 0.9643 | | - | - |
| logit(risk_2_) | 0.9643 | | - | - |
| logit(risk_3_) | 0.9653 | | - | - |
| Average length of 95% CI | |  | |  |
| logOR comparing adjacent levels | 0.2582 | | - | - |
| logRR_1_ | 0.2579 | | 0.7124 | 0.7130 |
| logRR_2_ | 0.5127 | | 0.8596 | 0.8627 |
| logRR_3_ | 0.7571 | | 0.7972 | 0.8027 |
| logit(risk_0_) | 1.9572 | | - | - |
| logit(risk_1_) | 1.9289 | | - | - |
| logit(risk_2_) | 1.9372 | | - | - |
| logit(risk_3_) | 1.9815 | | - | - |

*The estimations of logRR_1_, logRR_2_, logRR_3_, logit(risk_0_), logit(risk_1_), logit(risk_2_) and logit(risk_3_) of our method are based on 9927 simulations. All the other parameters are based on 10000 simulations.

| Two binary exposures with the disease prevalence=0.05 | Methods | | | |
| --- | --- | --- | --- | --- |
|  | The present method | | Sato | Miettinen |
| Estimate [true value] |  | |  |  |
| logOR_1_ [0.9163] | 0.9204 | | - | - |
| logOR_2_ [1.0986] | 1.1008 | | - | - |
| logRR_10_ [0.8862] | 0.8900 | | 0.8904 | 0.8906 |
| logRR_01_ [1.0588] | 1.0608 | | 1.0614 | 1.0614 |
| logRR_11_ [1.8908] | 1.8960 | | 1.8982 | 1.8995 |
| logit(risk_00_) [-3.8748] | -3.8981 | | - | - |
| logit(risk_10_) [-2.9585] | -2.9777 | | - | - |
| logit(risk_01_) [-2.7762] | -2.7973 | | - | - |
| logit(risk_11_) [-1.8599] | -1.8770 | | - | - |
| Variance ($\times100$) |  | |  |  |
| logOR_1_ | 1.9572 | | - | - |
| logOR_2_ | 1.8345 | | - | - |
| logRR_10_ | 1.8185 | | 3.6669 | 3.7229 |
| logRR_01_ | 1.7095 | | 2.7735 | 2.8251 |
| logRR_11_ | 3.5205 | | 3.5881 | 3.8089 |
| logit(risk_00_) | 5.3990 | | - | - |
| logit(risk_10_) | 5.6931 | | - | - |
| logit(risk_01_) | 5.1094 | | - | - |
| logit(risk_11_) | 5.7275 | | - | - |
| Coverage probability of 95% CI | |  | |  |
| logOR_1_ | 0.9488 | | - | - |
| logOR_2_ | 0.9512 | | - | - |
| logRR_10_ | 0.9493 | | 0.9484 | 0.9493 |
| logRR_01_ | 0.9500 | | 0.9505 | 0.9502 |
| logRR_11_ | 0.9476 | | 0.9473 | 0.9453 |
| logit(risk_00_) | 0.9509 | | - | - |
| logit(risk_10_) | 0.9522 | | - | - |
| logit(risk_01_) | 0.9549 | | - | - |
| logit(risk_11_) | 0.9516 | | - | - |
| Average length of 95% CI | |  | |  |
| logOR_1_ | 0.5462 | | - | - |
| logOR_2_ | 0.5308 | | - | - |
| logRR_10_ | 0.5264 | | 0.7447 | 0.7508 |
| logRR_01_ | 0.5118 | | 0.6510 | 0.6562 |
| logRR_11_ | 0.7282 | | 0.7343 | 0.7530 |
| logit(risk_00_) | 0.8964 | | - | - |
| logit(risk_10_) | 0.9238 | | - | - |
| logit(risk_01_) | 0.8819 | | - | - |
| logit(risk_11_) | 0.9325 | | - | - |

| Two binary exposures with the disease prevalence=0.01 | Methods | | | |
| --- | --- | --- | --- | --- |
|  | The present method | | Sato | Miettinen |
| Estimate [true value] |  | |  |  |
| logOR_1_ [0.9163] | 0.9197 | | - | - |
| logOR_2_ [1.0986] | 1.1034 | | - | - |
| logRR_10_ [0.9105] | 0.9138 | | 0.9113 | 0.9114 |
| logRR_01_ [1.0909] | 1.0958 | | 1.0941 | 1.0940 |
| logRR_11_ [1.9900] | 1.9980 | | 2.0011 | 2.0015 |
| logit(risk_00_) [-5.5480] | -5.6597 | | - | - |
| logit(risk_10_) [-4.6317] | -4.7400 | | - | - |
| logit(risk_01_) [-4.4494] | -4.5561 | | - | - |
| logit(risk_11_) [-3.5331] | -3.6365 | | - | - |
| Variance ($\times100$) |  | |  |  |
| logOR_1_ | 1.9557 | | - | - |
| logOR_2_ | 1.8654 | | - | - |
| logRR_10_ | 1.9238 | | 4.0051 | 4.0184 |
| logRR_01_ | 1.8425 | | 3.0492 | 3.0586 |
| logRR_11_ | 3.8777 | | 3.9444 | 3.9709 |
| logit(risk_00_) | 26.3264 | | - | - |
| logit(risk_10_) | 26.6204 | | - | - |
| logit(risk_01_) | 25.9008 | | - | - |
| logit(risk_11_) | 26.3623 | | - | - |
| Coverage probability of 95% CI | |  | |  |
| logOR_1_ | 0.9496 | | - | - |
| logOR_2_ | 0.9493 | | - | - |
| logRR_10_ | 0.9501 | | 0.9508 | 0.9504 |
| logRR_01_ | 0.9486 | | 0.9520 | 0.9509 |
| logRR_11_ | 0.9487 | | 0.9490 | 0.9494 |
| logit(risk_00_) | 0.9695 | | - | - |
| logit(risk_10_) | 0.9687 | | - | - |
| logit(risk_01_) | 0.9675 | | - | - |
| logit(risk_11_) | 0.9690 | | - | - |
| Average length of 95% CI | |  | |  |
| logOR_1_ | 0.5423 | | - | - |
| logOR_2_ | 0.5317 | | - | - |
| logRR_10_ | 0.5385 | | 0.7796 | 0.7808 |
| logRR_01_ | 0.5280 | | 0.6815 | 0.6825 |
| logRR_11_ | 0.7704 | | 0.7760 | 0.7798 |
| logit(risk_00_) | 1.9618 | | - | - |
| logit(risk_10_) | 1.9731 | | - | - |
| logit(risk_01_) | 1.9526 | | - | - |
| logit(risk_11_) | 1.9737 | | - | - |

*The estimations of logRR_10_, logRR_01_, logRR_11_, logit(risk_00_), logit(risk_10_), logit(risk_01_) and logit(risk_11_) of our method are based on 9930 simulations. All the other parameters are based on 10000 simulations.
